# Occurrence of Acute Oesophageal Necrosis (Black Oesophagus) in a Single Tertiary Centre

**DOI:** 10.3390/jcm8101532

**Published:** 2019-09-24

**Authors:** Orlando Davide, Carabotti Marilia, Ruggeri Maurizio, Esposito Gianluca, Corleto Vito Domenico, Di Giulio Emilio, Annibale Bruno

**Affiliations:** Medical-Surgical Department of Clinical Sciences and Translational Medicine, Sant’Andrea Hospital, University Sapienza, 00189 Rome, Italy; davidegiuseppeorlando@hotmail.it (O.D.); mariliacarabotti@gmail.com (C.M.); maurizio.ruggeri@gmail.com (R.M.); gle.esposito@gmail.com (E.G.); vito.corleto@gmail.com (C.V.D.); edigiulio52@gmail.com (D.G.E.)

**Keywords:** black oesophagus, acute oesophageal necrosis, gastrointestinal bleeding, upper gastrointestinal endoscopy, oesophageal stenosis, Zollinger–Ellison syndrome

## Abstract

Acute oesophageal necrosis (AON) is a rare condition characterised by the endoscopic finding of diffuse, circumferential, black mucosal pigmentation of the oesophagus, which typically stops at the gastro-oesophageal junction. This observational study aimed to assess the occurrence, clinical characteristics and outcomes of AON in a consecutive endoscopic cohort in a single tertiary university centre. A retrospective analysis of endoscopic data of upper gastrointestinal endoscopy (UGE) was carried out from 2008 to 2018. Out of 25,970 UGE, 16 patients (0.06%) had AON; 75.0% were men with a median age of 75 years. Almost all patients underwent diagnosis during emergency UGE performed for gastrointestinal bleeding, but one patient was diagnosed during elective UGE for persistent vomiting and diarrhoea. All patients reported one or more pre-existing comorbidities and concomitant acute events. Two patients had AON as the first presentation of Zollinger–Ellison syndrome (ZES). One patient developed an oesophageal stenosis, and another patient presented a relapse of AON. Mortality was 50%, but no patient died as a direct consequence of AON. AON is a rare cause of gastrointestinal bleeding diagnosed mainly during emergency UGE. Our study showed that ZES might manifest with this critical presentation, and endoscopists must be aware of this evidence.

## 1. Introduction

Acute oesophageal necrosis (AON), so-called black oesophagus, is a rare condition characterised by the endoscopic finding of diffuse, often circumferential, black mucosal pigmentation of the oesophagus, which typically stops at the gastro-oesophageal junction [1]. 

Epidemiological data of AON are scant, and the prevalence of this condition is supposed to be low. In fact, large retrospective series reported that the estimated incidence ranged from 0.01% [2,3] to 0.28% [4]_._

AON is more frequent in elderly men with medical comorbidities, often presenting with upper gastrointestinal bleeding, ranging from haematemesis, coffee ground emesis, and melena [1,5,6]. Prognosis is poor, with an overall mortality of 32% usually related to the underlying comorbidities and an estimated specific mortality of 6% [1,5,6].

The AON aetiology is likely multifactorial. It has been hypothesized that AON arises from a combination of oesophageal hypoperfusion with consequent ischaemia, impaired mucosal defence barrier and injury related to reflux of gastric contents [2]. To date, there has been no specific therapeutic protocol for this condition, and medical management has been directed to treat the coexisting underlying illness.

The aim of this observational retrospective study was to assess the occurrence, clinical characteristics and outcomes of AON in a consecutive endoscopic cohort over an 11-year period in a tertiary university centre.

## 2. Materials and Methods

A retrospective analysis of endoscopic data of elective and emergency upper gastrointestinal endoscopy (UGE) was carried out from January 2008 to December 2018 in a single tertiary university centre. Our electronic endoscopic database (Endobase Olympus) was searched using the following keywords: “oesophagus”, “black”, “necrosis”, and “necrotic”.

The criterion for the diagnosis of AON was the presence of circumferential black appearance of oesophageal mucosa that stops abruptly at the gastro-oesophageal junction [1,2,3,4,7]. Patients with other causes of oesophageal injury, such as caustic ingestion, severe esophagitis, malignancies and other benign disorders, were excluded. Gastric and duodenal endoscopic findings were assessed in all AON cases. Duodenal involvement-associated AON was considered in the presence of erosions, ulcers or necrotic aspects of the mucosa of the bulb and of the second portion.

Clinical records, including demographic, laboratory findings, radiological exams, pharmacological history, medical treatment, and outcome, were collected; each patient comorbidity was reported and expressed using the Charlson Index [8]. Anaemia was considered severe, moderate or mild when haemoglobin levels were ≤ 12–13, 8–10, and ≤ 8 g/dL, respectively [9].

Data were expressed as the percentage (%) of the total, or the median and interquartile range IQR or a range. The study was approved by the local ethics committee. 

## 3. Results

**Figure 1:**
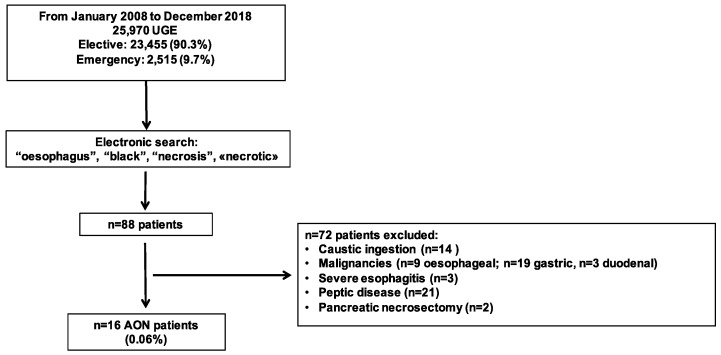
Flow-chart of the study. UGE, upper gastrointestinal endoscopy; AON, acute oesophageal necrosis.

According to the electronic search, 88 (0.34%) patients out of 25,970 were found in our endoscopic database. All endoscopic reports were individually checked to carefully ascertain the AON diagnostic criteria. Of the 88 patients, 72 were excluded, and 16 patients were diagnosed with AON (Figure 1). Almost all AON diagnoses were performed during emergency UGE [15/2515 (0.60%)], and only one case of AON was found during an elective exam [1/23,455 (0.004%)].

### 3.1. Demographics, Clinical Presentation and Comorbidities 

Of the 16 patients, 12 (75.0%) were men with a median age of 75 years (range 47–89).

The reasons for hospitalization were as follows: nine patients with upper gastrointestinal bleeding (56.2%), two patients with abdominal pain (12.5%), one patient with fever, one with persistent vomiting and diarrhoea, one with acute renal failure, one with traumatic fracture and one with dysphagia. The only case of AON observed during an elective endoscopy was admitted for persistent vomiting and diarrhoea. 

Clinical and laboratory data at the time of endoscopy, with indications for UGE, are reported in Table 1. 

Haemodynamic instability (hypotension and tachycardia) was present in three patients, one of which also presented hypoxemia. Physical examination of the other 13 AON patients was unremarkable. Thirteen patients (81.2%) had leukocytosis [median white blood cell count 14.800/µL (IQR = 13.000–18.700)], and 12 patients (75%) had elevated C-reactive protein [median 2.9 mg/dL (IQR = 2–12.3)]. Eleven patients (68.7%) had anaemia, and twelve (75%) had an elevated creatinine level [median 1.3 mg/dL (IQR = 1.9–5.4)], but a known history of chronic renal failure was reported only in five patients. Thirteen patients (81.2%) showed hyperglycaemia levels [median 185 mg/dL (IQR = 113–278)], but only seven of them were diabetic. Ten patients had low levels of albumin [median 2.8 g/dL (IQR = 2.1–3.6)].

All patients reported one or more concomitant pre-existing comorbidities: seven patients (43.7%) had diabetes, six (37.5%) had arterial hypertension, five (31.2%) had chronic renal failure, three (18.7%) had chronic ischaemic heart disease, three (18.7%) cerebral vascular disease and two (12.5%) had active malignancies (pancreatic adenocarcinoma). Concomitant acute events at the time of AON diagnosis were reported in almost all patients: seven (43.7%) had pneumonia, six (37.5%) had acute renal failure, two (12.5%) had Clostridium difficile colitis, one had acute myocardial infarction and one developed AON a few days after orthopaedic surgery. Two patients had AON as the first presentation of Zollinger–Ellison syndrome (ZES). Pre-existing and concomitant comorbidities in each patient are fully reported in Table 2.

### 3.2. Endoscopic Findings

AON was involved the entire length of oesophagus in seven patients (43.8%), the distal one-third in five (31.2%) and the distal two-thirds in four (25%) patients. Concomitant duodenal abnormalities were reported in 12 patients (75%): eight (50%) presented frank duodenal ulcers, two patients presented duodenal erosions, and the other two patients presented a necrotic aspect of the duodenum. Further endoscopic findings included seven cases of hiatal hernia, one case of Mallory–Weiss lesion, one case of gastric volvulus and one patient presenting necrotic aspect of the pylorus. 

**Figure 2:**
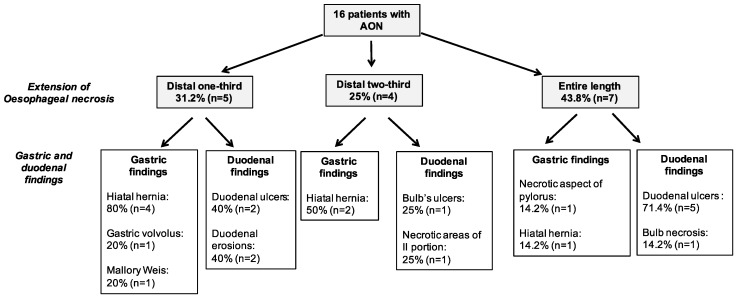
Endoscopic findings in acute oesophageal necrosis patients. AON, acute oesophageal necrosis.

**Figure 3:**
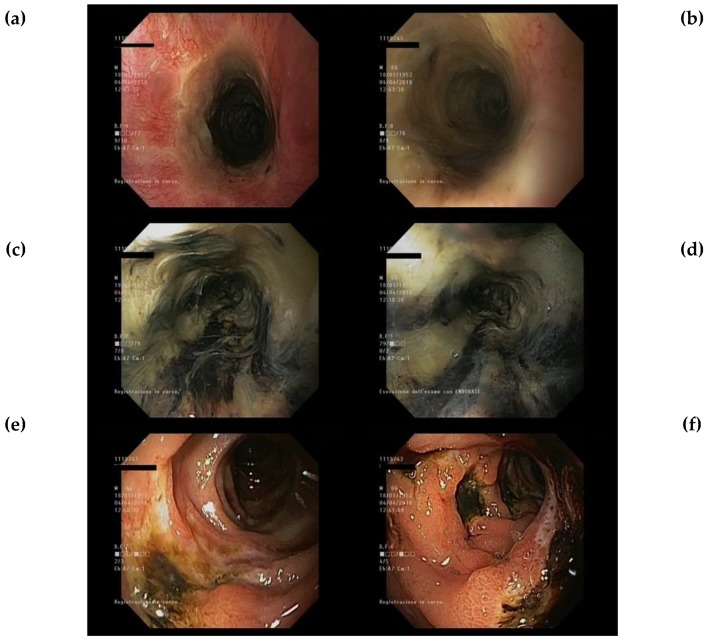
Endoscopic presentation of patient with AON. (**a**) Proximal oesophagus; (**b**) Proximal oesophagus; (**c**) Medium oesophagus; (**d**) Distal oesophagus; (**e**) Duodenal ulcers; (**f**) Duodenal ulcers.

Endoscopic gastric and duodenal findings with respect to oesophageal necrosis extension are reported in Figure 2. Endoscopic presentation of a patient with AON is showed in Figure 3. 

### 3.3. Medical and Endoscopic Treatment

Medical treatment for AON included intravenous proton pump inhibitors in all patients (81.2% continuous infusion for at least 72 h; 18.8% with twice a day intravenous administration). Nil per os and total parenteral nutrition were prescribed in 60% of patients. The majority of patients received antibiotics (73%) with associated antimycotics (25%). Eight patients (50%) required 1–4 red blood cell transfusions during hospitalization and two somatostatin infusions.

Among the twelve patients with duodenal involvement, three patients required endoscopic haemostasis due to ulcer bleeding. One patient further required transcatheter angiographic embolization and surgery to treat bleeding from duodenal ulcers.

### 3.4. Outcome

None of the patients developed oesophageal perforation. Specifically, in only nine cases, contrast-enhanced computed tomography CT was performed within 24 h of AON diagnosis, showing oesophageal thickening in five patients and duodenal thickening in three patients.

Eight patients (50%) died between 1–30 days of an AON event: three (37.5%) died from multi-organ failure, three (37.5%) died from septic shock, and two patients (25%) died from acute renal failure. None of the patients who died from septic shock had CT signs of perforation. 

**Figure 4:**
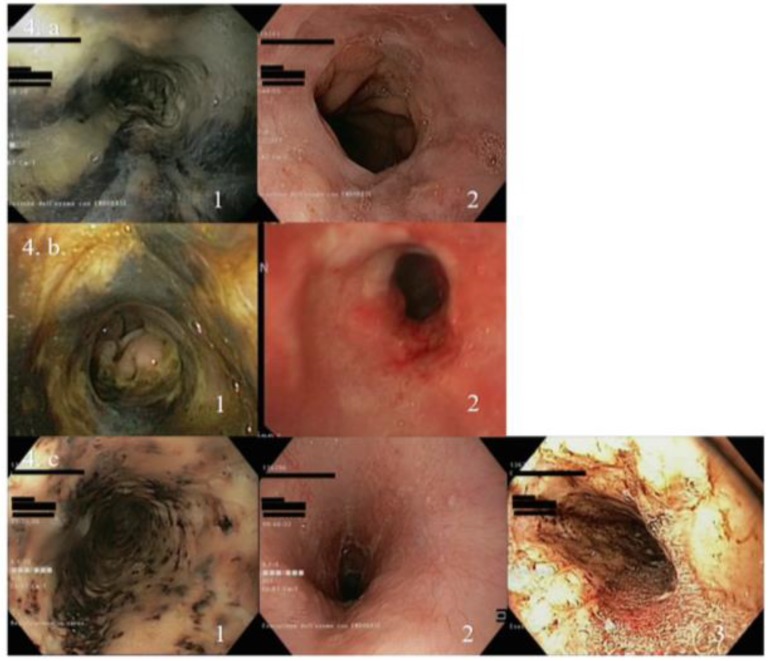
Endoscopic presentation of AON patients. (**a**) Endoscopic presentation of AON patient with remission. 1. AON at diagnosis; 2. AON remission after 51 days. (**b**) Endoscopic presentation of AON patient developing oesophageal stenosis. 1. AON at diagnosis; 2. Oesophageal stenosis. (**c**) Endoscopic presentation of patient who presented AON recurrence. 1. AON, endoscopic presentation at first episode; 2. AON remission after 15 days; 3. AON recurrence after 3 weeks.

At follow-up endoscopy, three patients showed AON remission, one patient developed oesophageal stenosis as an AON complication, and one patient complaining of vomiting and diarrhoea presented an AON relapse three weeks after the first episode (Figure 4). Notably, patients with AON recurrence are diagnosed with ZES.

Two patients completed uneventful hospitalization and were discharged without follow-up endoscopy. One patient was transferred to another hospital, and he was lost to follow-up.

The two patients with ZES underwent UGE twenty-eight months after the first AON episode, without showing any significant endoscopic findings.

## 4. Discussion

This study assessed the occurrence of AON in a consecutive endoscopic cohort over an 11-year period in a tertiary university centre. To the best of our knowledge, only a few case reports and case series describing AON are available, and the prevalence of this condition is still unknown.

Our study showed that AON is a very rare condition with a prevalence of 0.06%. Retrospective reports published more than twenty years ago reported an incidence that varies between 0.01% and 0.28% [2,3,4]; these data are quite similar compared to our report, further indicating the rare occurrence of AON.

The current study showed some interesting data. First, in our AON cohort, two patients presented a diagnosis of ZES. To our knowledge, this is the first time that AON has been reported as the first presentation of ZES; this represented a very interesting association, suggesting the possibility that some factors (i.e., acid hypersecretion) might contribute to the chemical and/or ischaemic injury of AON. Erosive oesophagitis has been frequently reported in patients with ZES; in a study by Hirschowitz et al., erosive oesophagitis was reported in 70% of ZES patients, with more than half of those patients having severe oesophagitis and 40% with complicated lesions (stricture, ulcer, or Barrett’s) [10]. On the other hand, an old report by Moreto et al. cited a single case of AON diagnosed after a surgical intervention of gastrinoma but unfortunately without reporting any additional patient data [3]. Although it is possible to speculate that hyperacidity might play an important role in the development of AON in these patients, it is possible that other specific triggers may act as concomitant mucosal injuries.

Second, we observed that one patient experienced an AON reoccurrence three weeks after the first event. This patient was affected by chronic ischaemic heart disease, hypertension and pneumonia and was subsequently diagnosed with ZES. AON reoccurrence is a rare event that has rarely been observed [11,12]. To our knowledge, only two other cases with AON relapse have been reported. Tanaka et al. reported the case of a male patient with multiple comorbidities (diabetes mellitus, hypertension, hyperlipidaemia, and angina pectoris) with a reoccurrence after approximately 5 months from the first episode [11], and Ramos et al. described a patient who developed a new AON after 4 months [12]. Even if recurrence is rare, it represents an important event to consider in this setting.

Third, we reported a patient with AON during an elective UGE performed for persistent vomiting and diarrhoea. This is a quite unusual clinical presentation since the majority of patients received a diagnosis of AON during an emergency UGE performed for gastrointestinal bleeding. However, in a recent case series, the case of a patient who incidentally received a diagnosis of AON during the endoscopic placement of a naso-gastrojejunal tube was reported [13], and in a previous report, an asymptomatic AON patient diagnosed during a percutaneous gastrostomy tube placement was described [5]. These atypical clinical presentations substantiated the hypothesis that AON might have a subclinical presentation, likely due to the short duration of the ischaemic/chemical damage that promoted spontaneous mucosal healing; on the other hand, it is possible that an early diagnosis has as counterpart a vague clinical scenario.

According to previous reports, our data show that AON mainly affected elderly male patients. We found that three-quarters of patients were male with a median age of 75 years, similar to other reports where men are four times more commonly affected than women, with a peak of incidence in the sixth or seventh decade of life [5,6]. In addition, we confirmed that AON patients have multiple chronic and overlapping acute comorbidities, presenting in the majority of cases with upper gastrointestinal bleeding. However, even if gastrointestinal bleeding (i.e., haematemesis and melena) is the indication to perform UGE in more than 80% of AON patients, haemodynamic instability was observed only in a few patients.

Regarding the endoscopic findings, we reported that AON involved the entire length of oesophagus in almost half of AON patients, mostly with concomitant duodenal findings.

Even if AON more frequently affected the distal one-third and the distal two-thirds of the oesophagus, Lamers et al. also reported panoesophageal involvement in a remarkable number of AON patients (three out of five) [13]. Similar to what has been previously reported, the extent of oesophageal involvement seemed to be related to the duodenal disease, with almost all patients with panoesophageal injury having a duodenal injury as ulcer and bulb necrosis. This phenomenon might be likely explained by the common blood supply from the celiac axis branches to the oesophagus and duodenum. The typical relative sparing of gastric mucosa might be explained by the different response to the acid insult, since compared to gastric mucosa, oesophageal and duodenal mucosa presented a slower healing process. In addition, duodenal lesions may result in gastric outlet obstruction that, in turn, potentiates the development of mucosal necrosis of the distal oesophagus [1,14].

Oesophageal perforation is the most serious AON complication, generally occurring in less than 7% of patients [5,6]; however, in our AON cohort, no case of perforation has been reported. On the other hand, we documented stenosis as a complication of AON in a patient with involvement of the distal two-thirds of the oesophagus. We found a prevalence of approximately 6%, which is quite similar to other cohorts in which the reported frequency of oesophageal stenosis has been reported in 10% of patients [5,6].

Then, we found a mortality rate of 50% with no patients who died as a direct consequence of AON. Even if no patient died from AON, the overall mortality seems slightly higher compared to previous studies: Augusto et al. showed a mortality rate of 34.5%, and none was a direct consequence of AON [4], whereas Gurvits et al. reported a 5.7% mortality rate due to AON and an overall mortality rate of 31.8% [4,5]. In a more recent study, the mortality rate was 12.5% and unrelated to AON [14]. Even if the specific AON mortality rate remained low, these overall mortality proportions might reflect the clinical severity of included patients in the specific cohort.

We are aware of some limitations of this study. The main limitation is the retrospective nature of the data collection. In addition, oesophageal biopsies were not always available; however, the diagnosis of AON is endoscopic, and oesophageal biopsies are supportive but not required to make the proper diagnosis. This AON cohort has been collected in a single tertiary university centre, which might affect the generalisability of the study’s results.

## 5. Conclusions

In conclusion, AON is a rare cause of gastrointestinal bleeding diagnosed mainly during emergency UGE, but it can diagnosed during elective endoscopy. Beyond the well-known association between AON and elderly patients with multiple comorbidities, our study showed that ZES might manifest with this critical presentation, and endoscopists must be aware of this evidence.

## Figures and Tables

**Table 1:** Clinical, laboratory data, and indications for UGE in AON patients (*n* = 16).

Physical Examination	
Hypotension (lower than 100/60 mmHg)	3/16 (18.7%)
Tachycardia (>100 bpm)	3/16 (18.7%)
Hypoxemia (SpO2 < 60)	1/16 (6.2%)
**Laboratory Analysis**	
Leukocytosis (WBC > 10.000/µL)	13/16 (81.2%)
Increased C-reactive protein (>0.5 mg/dL)	12 /16 (75%)
Anaemia	11/16 (68.7%)
Mild	6/11 (54.5%)
Moderate	2/11 (18.2%)
Severe	3/11 (27.3%)
Increased glycaemia (>100 mg/dL)	13/16 (81.2%)
Increased creatinine (>1.5 mg/dL)	12 /16 (75%)
Hypoalbuminemia (<3 g/dL) *	10/11 (90.9%)
**Indications for UGE**	
Upper GI bleeding	13/16 (81.2%)
Vomiting	1/16 (6.2%)
Worsening of anaemia	1/16 (6.2%)
Dysphagia	1/16 (6.2%)

Bpm = beats per minute; UGE = upper gastrointestinal endoscopy; GI = gastrointestinal; WBC = White blood cell. * Albumin dosage was available in 11 patients.

**Table 2:** Demographic characteristics, comorbidities, endoscopic findings and outcome of patients with AON.

Year of Diagnosis	Age	Gender	Chronic Comorbidities	Acute Concomitant Comorbidities	Charlson Index	Oesophageal Necrosis Extension	Duodenal Involvement	Other Endoscopic Findings	Outcome
2008	74	M	Chronic renal failure, diabetes, chronic ischaemic heart disease and cerebral vascular disease	Acute renal failure	5	I-II-III	Duodenal’s ulcers	-	Discharged without follow-up endoscopy
2009	76	M	Cerebral vascular disease	Pneumonia and sepsis	3	I-II-III	Duodenal’s ulcers	-	Death from septic shock
2010	80	M	Chronic renal failure	Pneumonia and acute renal failure	5	I-II-III	Bulb’s necrosis	Necrotic aspect of pylorus	Death from multi-organ failure
2010	74	M	Chronic renal failure, dilatative cardiomyopathy and COPD	Acute renal failure	7	I-II-III	-	-	Lost to follow-up
2012	47	M	Diabetes, atrial fibrillation and psoriasis	Pneumonia	3	II- III	-	Hiatal hernia	Oesophageal stenosis
2012	69	M	Arterial hypertension, diabetes and depressive syndrome	Vertebral fracture and CD colitis	3	III	Duodenal’s ulcers	Hiatal hernia Mallory Weiss	Discharged without follow-up endoscopy
2014	81	M	Chronic renal failure, silicosis and brachy-tachycardia syndrome	Pneumonia	6	I-II-III	Duodenal’s ulcers	-	Death from multi-organ failure
2016	64	M	IV stage pancreatic adenocarcinoma	Recent NSAID assumption	8	II-III	Bulb’s ulcers	Hiatal hernia	Death from acute renal failure
2016	65	F	Zollinger–Ellison syndrome in metastatic NET	Vomiting and diarrhoea	8	I-II-III *	Duodenal’s ulcers	Hiatal hernia	Remission at follow-up UGE
2016	85	M	Alzheimer disease, decubitus lesions and atrial fibrillation	Pneumonia and CD colitis	5	III	Duodenal’s erosions		Death from septic shock
2016	84	F	Zollinger–Ellison syndrome in pancreatic gastrinoma, chronic ischaemic heart disease and arteria hypertension	Pneumonia	12	III	Duodenal’s ulcers	Hiatal hernia	AON relapse 3 weeks after the first episode
2017	87	F	Pancreatic adenocarcinoma, chronic ischaemic heart disease, hypertension, diabetes, Parkinson disease and senile dementia	Acute renal failure	9	III	Duodenal’s erosions	Hiatal hernia	Death from acute renal failure
2018	71	M	Diabetes	Superficial thrombophlebitis	4	II- III	-	-	Remission at follow-up UGE
2018	89	M	Hypertension, diabetes, dyslipidaemia and glaucoma	Pneumonia	5	III	-	Hiatal hernia gastric volvolus	Death from septic shock
2018	66	M	Charcot Marie Tooth polyneuropathy, arterial hypertension and diabetes	Acute renal failure	3	I-II-III	Duodenal’s ulcers	-	Remission at follow-up UGE
2018	77	F	Cerebral vascular disease, Alzheimer disease, arterial hypertension and chronic renal failure	NSTEMI and acute on chronic renal failure	7	II-III	Necrotic areas of II portion	-	Death from multi-organ failure

M, male; F, female; NET, neuroendocrine tumour; CD, *Clostridium difficile*; NSAID, nonsteroidal anti-inflammatory drug; COPD, chronic obstructive pulmonary disease; UGE, upper gastrointestinal endoscopy; III, distal one-third; II, distal two-third; I, proximal one-third; * Elective endoscopy.
